# Epidemiological consequences of household-based antiviral prophylaxis for pandemic influenza

**DOI:** 10.1098/rsif.2012.1019

**Published:** 2013-04-06

**Authors:** Andrew J. Black, Thomas House, M. J. Keeling, J. V. Ross

**Affiliations:** 1 Stochastic Modelling Group, School of Mathematical Sciences, The University of Adelaide, Adelaide, South Australia 5005, Australia; 2 Warwick Mathematics Institute, University of Warwick, Coventry CV4 7AL, UK; 3 School of Life Sciences, University of Warwick, Coventry CV4 7AL, UK

**Keywords:** A(H1N1)pdm, Bayesian, doubling time, Markov chain, R_*_

## Abstract

Antiviral treatment offers a fast acting alternative to vaccination; as such it is viewed as a first-line of defence against pandemic influenza in protecting families and households once infection has been detected. In clinical trials, antiviral treatments have been shown to be efficacious in preventing infection, limiting disease and reducing transmission, yet their impact at containing the 2009 influenza A(H1N1)pdm outbreak was limited. To understand this seeming discrepancy, we develop a general and computationally efficient model for studying household-based interventions. This allows us to account for uncertainty in quantities relevant to the 2009 pandemic in a principled way, accounting for the heterogeneity and variability in each epidemiological process modelled. We find that the population-level effects of delayed antiviral treatment and prophylaxis mean that their limited overall impact is quantitatively consistent (at current levels of precision) with their reported clinical efficacy under ideal conditions. Hence, effective control of pandemic influenza with antivirals is critically dependent on early detection and delivery ideally within 24 h.

## 1. Introduction

Despite its relative mildness, the 2009 influenza pandemic was still a significant cause of mortality and morbidity. The potential for future severe pandemics continues to present a major threat [1]. Faced with a virulent strain of pandemic influenza, one of the main public-health objectives is to control or contain the outbreak for sufficiently long that a vaccine can be developed. Treatment with antivirals offers the potential to enable such control. The fundamental policy is to give antiviral treatment to all household members (or other close contacts) as soon as an infection is identified within the household [2–4]. This has several aims: it lowers the risk of onward transmission from both those currently infected and from subsequent household cases, and it decreases the severity and duration of disease (both for those already infected and for subsequent household cases).

Clinical trials of the two major antiviral treatments against influenza, oseltamivir and zanamivir, have shown subtly different effects. Both treatments appear to have similar effects in lowering susceptibility to infection, but oseltamivir appears to be more effective in reducing transmission from treated infected individuals [5,6]. However, a fundamental challenge is to link these individual-level observations to population-level predictions about the effectiveness of this type of treatment. This is precisely the type of complex problem where multiple scales and nonlinear behaviour mean that mathematical models are essential tools.

While detailed, large-scale simulation models of entire populations are now feasible [7], the computational requirements of such models precludes the number of replications necessary to perform wide-ranging sensitivity analysis. In contrast, while simple models based on homogeneously mixing populations can be efficiently used, they do not admit sufficient complexity to capture the localized correlation between detection of disease and treatment with antivirals. Household models offer a compromise, in which great computational efficiency can be achieved, and yet the household-level distribution of antiviral treatment (both reactively and prophylactically) can be robustly modelled. Deterministic and stochastic household models have been considered in the literature [8,9]; herein we focus on (discrete-state) stochastic models, as most appropriate for the very early stages of an epidemic.

Household models are an increasingly popular framework for studying disease dynamics [9–11]. These models capture the epidemiological observation that a small number of household contacts are responsible for a significant amount of transmission, and that such contacts are highly clustered forming a clique. Such models are also the simplest available that contain the necessary population heterogeneity required to accurately model antiviral prophylaxis, robustly capturing the fact that antivirals are generally administered to entire households. The small number of individuals within a household additionally means that the chance nature of transmission (and recovery) is likely to be influential, and therefore models must allow for the stochastic aspects of epidemic processes. One further advantage of this approach is that parametrization through likelihood calculation becomes feasible [9,10,12,13]; although it is possible using Monte Carlo simulations, in practice this is likely to be too slow.

Here, we introduce a general modelling framework for infectious disease dynamics in a population of households, allowing for complex control interventions, focusing specifically on the impact of household antiviral treatment. Given the computational efficiency of this methodology, we are able to fully explore the ranges of uncertainty in the effects of treatments, and pay considerable attention to the impact of delays between detection and deployment of the treatments. Two specific scenarios concerning this delay are considered: in the most general form, we allow for random detection delay (for each infected member of the household) to the notification of authorities of possible infection, and then subsequent random deployment delay until intervention is begun; we also consider the specific case of a fixed delay to intervention following the first infectious case within the household.

Two simple measures are used to capture the population-level transmission effects of any treatment regime: the household basic reproduction number, R_*_, which measures the average number of secondary households infected by a household when the clear majority of households are fully susceptible [9] and the doubling time early in the pandemic, *T*_d_. To calculate these, we extend the computationally efficient methods recently presented for evaluating these characteristics in a model with a homogeneous distribution of household sizes [10] to the higher dimensional case of a heterogeneous distribution of household sizes based upon census data. We use census data from Indonesia (2003), the UK (2001) and Sudan (1990), providing a contrast between populations dominated by single and two-person households to ones where households of size four and larger are most common. Throughout this paper, our default assumptions and parameters used are based on the 2009 H1N1 pandemic, although we believe our results should translate to other influenza outbreaks.

In common with many methods of control and other studies [2–4,14–19], we discover that prompt action is as important as effective action; that is to contain a pandemic, even a highly efficacious antiviral treatment must be administered rapidly. Nevertheless, delayed household antiviral treatment can still significantly increase the doubling time of the epidemic, buying time for other control measures.

### 1.1. Relation to previous work

Before 2009, many modelling papers were published that dealt with mitigation of pandemic influenza, mainly motivated by concerns about H5N1 strains emerging from southeast Asia [3,4,15–19]. This work was typically based on computationally intensive Monte Carlo simulation using estimates of parameters from diverse sources, together with traditional sensitivity analysis—although due to the complexity of the models only a few realizations were generally possible. In these models, a number of control measures were simultaneously simulated with the aim of containing an outbreak of a highly virulant strain as rapidly as possible. As such, these provided important general guidance to public-health policy planning, which by necessity involves multiple interventions.

The motivation for our current work is different—we wish to make a careful quantitative assessment of one particular intervention (antivirals) to address the seeming discrepancy between the efficacy of this intervention in clinical trials and its lack of major impact at the population level during the 2009 pandemic. We therefore focus on a simpler households model, as has been considered in more theoretical modelling work [20–22], with two levels of mixing only—within and between households. This analysis can be performed with extreme computational efficiency; in fact, we circumvent the need for simulation of model outputs (given parameter values), instead evaluating our epidemiological quantities to a desired numerical precision.

This computational efficiency allows us to achieve the methodological ideal of fully accounting for uncertainty in parameter values. We use posterior distributions for all parameters, estimated from antiviral meta-analysis [6] and influenza A(H1N1)pdm09 transmission data [23,24] and report full kernel density estimates, along with credible intervals, for all our results. Although we focus on relatively simple models, much more epidemiological detail could easily be included within the general framework (for example, asymptomatic individuals). In this work, we have only included aspects which can be robustly parametrized. As such our results provide novel quantitative insights into the impact of antivirals on the 2009 pandemic and add to the ongoing debate concerning antiviral efficacy [25].

## 2. Models

In the basic household modelling framework, there exist two levels of transmission: strong transmission between members of the household, and weaker transmission between individuals from different households. Typically households have a small number of individuals so the internal dynamics must be modelled stochastically. In this study, we are primarily concerned with modelling pandemic influenza, so we use an SEEIIR (*susceptible–exposed–infected–recovered* with two exposed and two infectious classes) model for the infection dynamics; this model has been used in a number of previous pandemic influenza studies [26,27]. The two stages in both the latent and infectious periods mean that these periods have an Erlang-2 distribution [10,28], which matches the observed transmission profile [29].

Within the household, infectious individuals can infect susceptible individuals via transmission that is assumed to be frequency dependent [30] in our investigations of the model reported in §3, while in our main investigation of pandemic influenza as reported in §4, we use an estimate of the transmission parameter for each household size. The transmission parameter is denoted *β_k_* in a household of size *k*. Newly infected individuals then pass through two latent and two infectious classes before recovering—the rates of progression through these classes, *σ* and *γ*, are independent of the household size and composition. The basic events that define the within-household model are detailed in table 1.

**Table 1.** The transitions and associated rates defining the stochastic SEEIIR model for the within-household dynamics; *k* is the size of the household. Only the states that change in a given transition are shown, all others remain constant. The parameters *τ* and *ρ* control the reduction in transmission and susceptibility when antivirals are administered to all members the household, hence *τ* = *ρ* = 0 for the uncontrolled epidemic.

event	transition	rate
internal infection	(*S*, *E*_1_) → (*S* − 1, *E*_1_ + 1)	*β_k_*(1 − τ)(1 − *ρ*)*S*(*I*_1_ + *I*_2_)/(*k* − 1)
latent progression	(*E*_1_, *E*_2_) → (*E*_1_ − 1, *E*_2_ + 1)	2*σE*_1_
start shedding	(*E*_2_, *I*_1_) → (*E*_2_ − 1, *I*_1_ + 1)	2*σE*_2_
infection progression	(*I*_1_, *I*_2_) → (*I*_1_ − 1, *I*_2_ + 1)	2*γI*_1_
recovery	*I*_2_ → *I*_2_ − 1	2*γI*_2_

**Figure 1.**
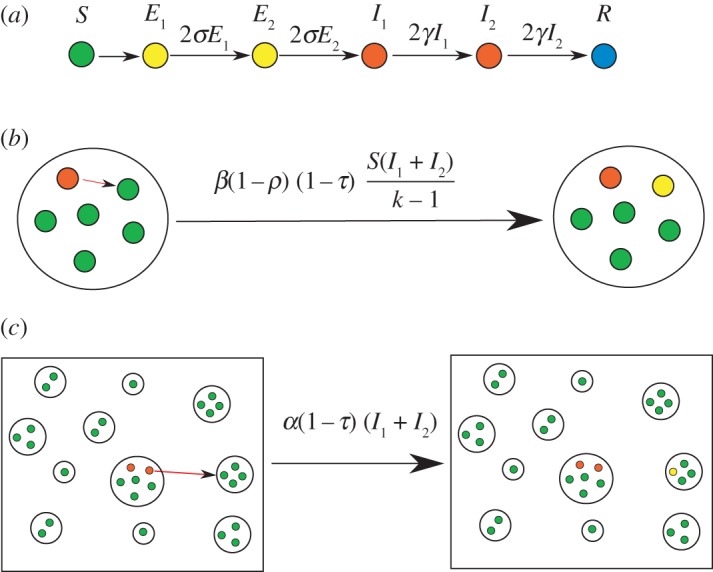
The basic household model used in this paper. There are three levels to this model: (*a*) the individual level, (*b*) the within-household level where there is strong mixing, and (*c*) the population level in which there is weaker mixing and a distribution of household sizes.

To maintain infection within the population, it is required that infection can spread between households. In particular, it is assumed that a susceptible individual contracting infection from outside their household occurs at a rate equal to *α* times the total prevalence of infection in the population. The basic structure of the model is illustrated in figure 1. To gain analytical traction on this model, we make the simplifying assumption that new infections outside a given household result in a naive household being infected, and hence that households are only ever infected once. Given that we are concerned with the early growth rate of an outbreak, this is a reasonable assumption which is asymptotically exact in the limit of an infinite population of randomly mixing households early in the epidemic. We compare this theoretical argument against Monte Carlo simulations for a range of population sizes in the electronic supplementary material.

For this study, we concentrate on quantities that capture the early epidemic behaviour: the household basic reproduction number, R_*_, and the doubling time early in the pandemic, *T*_d_. The household basic reproduction number, R_*_, is the equivalent of the more familiar epidemiological measure of *R*_0_ [31], but captures the expected number of secondary households infected in the early stages of an epidemic [9,10]. It should be stressed that R_*_ is a population-level threshold [9].

We first demonstrate how these values can be calculated for a heterogeneous distribution of households, assuming no interventions, in terms of the expectation of a path integral of a Markov chain. This generalizes the computationally efficient methodology first introduced in [10]. Later, we consider how these quantities are modified when antiviral interventions are also included. We provide Matlab code to implement this methodology via the *EpiStruct* project [32].

### 2.1. Early dynamics for heterogeneous distribution of household sizes

In the study of Ross *et al*. [10], efficient methods were presented for evaluating a number of characteristics of the dynamics of infection in a population of interacting households. Here we extend this methodology to the realistic scenario in which we have a heterogeneous distribution of household sizes *h_k_*, where *h_k_* is the proportion of households of size *k*.

An important distribution for our results is the *size-biased distribution*, *π_k_*:2.1
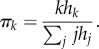


This is the probability that a randomly selected individual from the population belongs to a household of size *k*.

The household basic reproduction number, R_*_, is defined as the expected number of secondary households infected by a single household with initially one infected member, when the population is completely susceptible. If *X_k_*(*t*) is the continuous-time Markov chain describing the infection dynamics for a household of size *k*, then we define the function *I*(*X_k_*(*t*)) as giving the number of infectious individuals in the household at time *t*. The household reproduction number is then given by,2.2
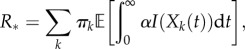
where the expectation of the integral in (2.2) may be evaluated by solving a system of linear equations for each household size *k*, as detailed in the study of Ross *et al*. [10]. The initial condition for the Markov chain, *X_k_*(0), is taken to be a single exposed individual in the first class *E*_1_, with all other individuals susceptible.

The early growth rate, *r*, which is the rate of exponential growth matching the expected early growth of the dynamic household disease model, is found by solving,2.3
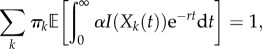
where the expectation of the integral, here with exponential discounting at rate *r*, may again be evaluated by solving a system of linear equations for each household size *k* [10]. This integral is then combined with a numerical root-finding method to determine *r*; here we have adopted Matlab's fzero routine throughout. The doubling time of the early epidemic, *T*_d_, is simply the time for the number of cases to double (a quantity that can often be robustly estimated from epidemic data as it is unaffected by constant additive or multiplicative errors) and is related to the early growth rate, *r*, by *T*_d_ = ln(2)/*r*.

### 2.2. Modelling antiviral interventions

In §2.1, we discussed how to calculate the household basic reproduction number, R_*_, and the early doubling time, *T*_d_, for a heterogeneous distribution of households in the absence of intervention. We now discuss the necessary modifications to the basic model in order to account for pharmaceutical interventions. The main challenge is modelling the delay between the introduction of the disease to a household and the allocation of antivirals to the household.

We assume the intervention, once it takes place, has two main outcomes. Firstly, it reduces the susceptibility of all individuals within the household to a fraction (1 − *ρ*) of their original susceptibility, where 0 ≤ *ρ* ≤ 1. Secondly, the intervention reduces the within- and between-household transmission rates to a fraction (1 − *τ*) of their original values, where 0 ≤ *τ* ≤ 1. A range of other assumptions are possible within our model framework (such as an increased recovery rate) but for influenza, our modelling assumptions (motivated by current knowledge [5,6]) are that the two effects represented by *τ* and *ρ* are sufficient to capture the important features of the system. The events and rates which define the model are summarized in table 1; pre-allocation *τ* = 0 and *ρ* = 0, and post-allocation *τ* > 0 and *ρ* > 0.

We consider three schemes to model the delay between the initial infection and the effects of intervention: a constant delay following the first infectious case within the household; an exponentially distributed delay; and finally, a delay to notification of possible infection presence within a household, followed by an exponentially distributed delay to intervention. The constant and exponentially distributed delays represent two relatively extreme cases. The scheme with notification involves each infectious individual within the household independently notifying authorities of their possible infectious status at some rate *r_n_*, and once notified, there exists an exponentially distributed delay to delivery and the effects of intervention as in the previous case. Throughout we focus on the average time from first symptoms to when the antivirals take effect, and term this the *mean delay*.

For these schemes, the household basic reproduction number R_*_ and early doubling time *T*_d_ can be calculated as in §2.1 using the extended Markov chains. For the case of constant delay, the expression for the expectations can be split into two parts, with different dynamics before and after the antivirals. Full mathematical details of all three schemes and the various calculations involved are given in the electronic supplementary material.

One of the central claims of this paper is the efficiency with which we can compute results. This can be seen by comparing times for computation of the path integrals with that of stochastic simulations. For example, on a 2.5 GHz Intel Core i5 machine running Matlab, the average time to evaluate equation (2.2) is 6.4 × 10*^−^*^3^ s (this assumes the exponential model with *k* = 1, … , 7). In contrast, 10^4^ replications of a Gillespie simulation of the same model takes 18 s. This represents a speed up of three orders of magnitude, thus large comprehensive sweeps of parameter space, such as those shown in this paper, are computationally infeasible using a naive Monte Carlo method.

## 3. Results

### 3.1. General behaviour of antivirals

**Figure 2.**
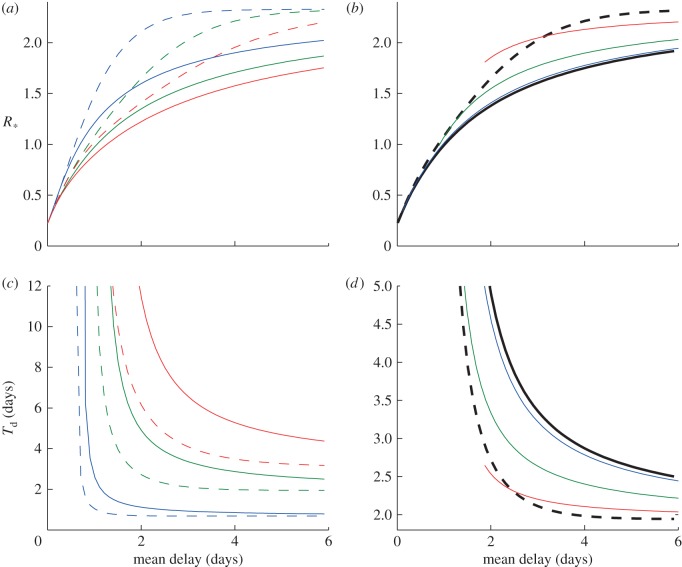
General behaviour of antivirals. Household basic reproduction number, R_*_, and doubling time early in the pandemic, *T*_d_, as a function of the mean delay to antiviral allocation for the SEEIIR model. The mean delay is taken as the time from the first infectious case to when antivirals take effect. This is composed of notification and further allocation delay. (*a*,*c*) SEEIIR with constant and exponential delay. (*b*,*d*) SEEIIR with notification (*σ* = 1). Dashed and solid lines are from the constant and exponential delay models, respectively, for three values of *σ*, where 1/*σ* is the average exposed period. (*b*) The coloured lines show the model results with notification for *σ* = 1. Black dashed and solid lines are constant and exponential delay models, respectively. The coloured curves end at minimum delay possible for a given value of the notification rate, *r_n_*. (*c*,*d*) The doubling time, using the same colour scheme. Other parameters: *k* = 3, *β_k_* = 2, *α* = 1, *γ* = 1, *τ* = 0.8 and *ρ* = 0.8. All rates are given in terms of days^–1^. (*a*,*c*) Blue lines, *σ* = 50; green lines, *σ* = 1; red lines, *σ* = 0.5. (*b*,*d*) Blue lines, *r*_n_ = 10; green lines, *r*_n_ = 1; red lines, *r*_n_ = 0.2.

To illustrate the dynamics, we compare the three intervention schemes for a homogeneous population of households of size *k* = 3. Figure 2 shows how R_*_ and *T*_d_ vary with the mean delay to intervention. Part A shows how R_*_ varies assuming constant (dashed lines) and exponentially distributed delays (solid lines) for three values of exposed period parameter *σ*. Part B shows the model results incorporating notification for a single value of *σ*, with the black lines representing the two extreme cases of a constant delay (dashed line) and exponentially distributed (solid line) delay. To maintain a consistent definition of mean delay, we add on the mean delay due to notification, which is why the coloured curves start at non-zero values for the mean delay; these initial values represent the minimum possible delay for a given value of *r_n_*. In the limit *r_n_* → ∞, corresponding to instantaneous notification, the notification curve tends to that of the exponential distribution without notification, as expected. The limit *r_n_* → 0 corresponds to households never notifying the authorities, and hence antivirals have no effect.

Figure 2*c*,*d* shows the corresponding early doubling time, *T*_d_, for the same set of models. We can see that the long exposed periods (smaller *σ*) have a large effect on the minimum doubling time *T*_d_, but not the basic reproduction number R_*_, irrespective of the mean delay. In all cases, the notification curves lie broadly within the limits of the constant and exponential delay cases hence we consider only these two extremes from now on.

### 3.2. Impact of demographics

**Figure 3.**
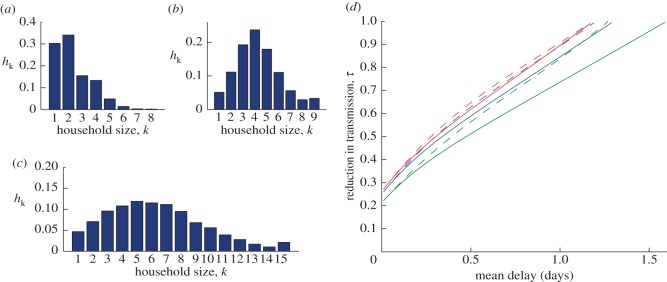
The reduction in transmission needed to reduce the household reproductive ratio, R_*_ < 1, for different household size distributions. (*a*–*c*) Three household size distributions for (*a*) UK 2001, (*b*) Indonesia 2003 and (*c*) Sudan 1990. The values of R_*_ for an uncontrolled epidemic are 2.3, 3.4 and 4.7 for the distributions (*a*–*c*), respectively. (*d*) The minimum value of the antiviral efficacy, *τ*, and the maximum mean delay to reduce R_*_ = 1 for the three distributions (*a*–*c*). The dashed lines are for the model assuming constant delay and solid lines are for the exponential delay (green, UK; blue, Indonesia; red, Sudan). Other parameters: *β_k_* = 2, *α* = 1, γ = 1, *σ* = 1 and *ρ* = 0.8. All rates are given in terms of days^–1^.

Figure 3*a*–*c* shows the household size distributions for the UK (2001), Indonesia (2003) and Sudan (1990). These were chosen to represent a range of distributions. Many western household size distributions, for example, those of USA and Australia, are very close to the UK data presented. To investigate the behaviour of the models incorporating distributions, we calculate the reduction in transmission, *τ*, needed to bring R_*_ = 1 as a function of the mean delay. Figure 3*d* shows this using the three different household size distributions and focusing on just constant and exponential delays. We see that the bias towards larger household sizes in both Indonesia and Sudan means that the maximum possible delay is smaller for a given value of *τ*, although the shift is not large.

## 4. Pandemic influenza model

### 4.1. Parametrization

We now consider the application of our methods to assess the use of antivirals to mitigate an outbreak of influenza, with appropriately estimated parameters and distributions. We focus in particular on the 2009 H1N1pdm outbreak. We estimate the parameters of our model in two stages. Firstly, we take a sample of 10 000 estimates for the transmission rate parameters from the posterior distribution of parameters estimated in [24]. This paper reports on the use of Bayesian statistical methods to estimate transmission probabilities stratified by household size, including probabilities describing case ascertainment, using data collected from 424 households in Birmingham, UK, during the first seven weeks of the 2009 H1N1 pandemic. As elsewhere in this paper, by using these estimates we are assuming that the observed pandemic was very close to what would happen in the absence of antivirals.

To estimate the latent and infectious periods for H1N1, we use data from the study of Donnelly *et al*. [23], which collates *clinical serial interval* data from seven epidemiological studies in the USA early in 2009. The clinical serial interval is the time between date of symptom onset in the index case and the date of symptom onset in one of its secondary cases. By computing the (theoretical) distribution of serial intervals for the SEEIIR model, we can then use these data and Bayesian MCMC methods to estimate a posterior distribution for *σ* and *γ*. Details of the calculation of the serial interval distribution for this model, and the Bayesian methodology, are given in the electronic supplementary material.

**Figure 4.**
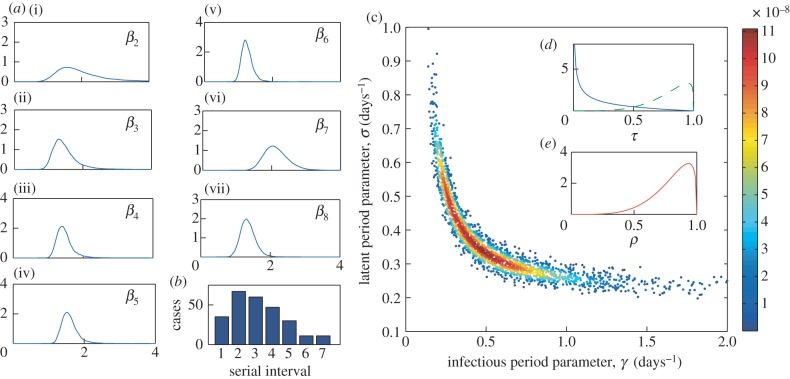
Pandemic influenza parameter estimates. (*a*(i–vii)) Kernel density plots for the within-household transmission rate, *β_k_*, from 10 000 random samples from the posterior distributions given in [24]; (*c*) 2000 (randomly selected from the 10 000) random samples for the posterior distribution for the infectious and latent period parameters, *γ* and *σ*, estimated by fitting to the serial interval data (*b*); points are coloured according to their likelihood value as per the scale on the right. (*d*,*e*) The posteriors for the reduction in transmission, *τ*, (for both zanamivir (blue) and oseltamivir (green)) and the reduction in susceptibility, *ρ*.

Figure 4*c* shows 2000 random samples from the posterior distribution estimated by fitting to the serial interval data provided in the study of Donnelly *et al*. [23] (also presented in figure 4*b*) using our methodology. The distributions for parameters *τ* and *ρ* are shown in figure 4*d,e*. These are beta density functions parametrized to match the mean and 95% confidence intervals from the antiviral studies reviewed in Halloran *et al*. [6]. The estimated reduction in transmission was significantly different for the two drugs zanamivir and oseltamivir, hence we provide two distributions for these. The reduction in susceptibility was approximately the same for both drugs. Finally, the between-household transmission rate was set as *α* = 1.18; this was chosen to give a doubling time of approximately 7 days in the absence of any interventions and is in line with estimates from the 2009 outbreak [33].

### 4.2. Results

We take 10 000 random samples of the parameters from the posterior and estimated distributions, each of which, via our methodology, provides a sample from the distribution of the household basic reproduction number, R_*_, and doubling time early in the epidemic, *T*_d_. We used Matlab's ksdensity routine to produce kernel posterior predictive densities of R_*_ and *T*_d_. This estimates a smooth probability density function from a finite sample of a random variable.

**Figure 5.**
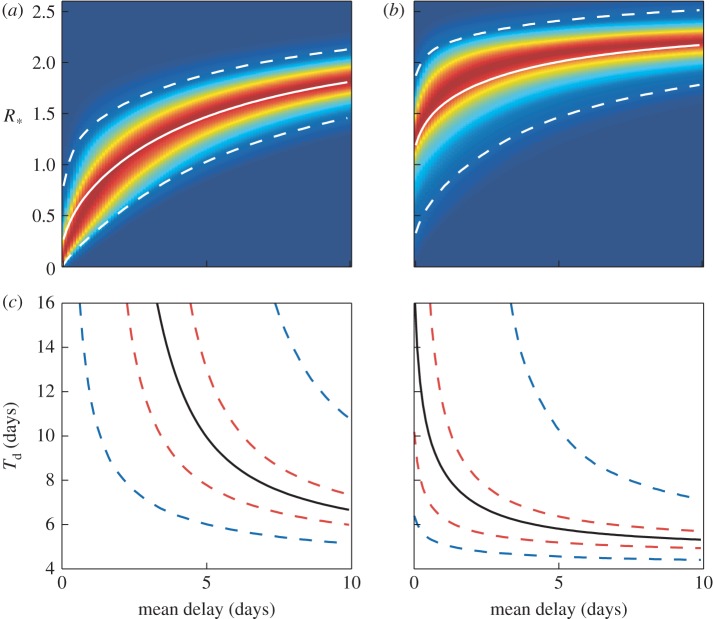
Pandemic model results for the exponentially delayed model. (*a*,*b*) Kernel density plots for the household reproductive ratio, R_*_, as a function of the mean delay to allocation for the two types of antivirals oseltamivir and zanamivir. Solid and dashed white curves in (*a*) and (*b*) mark the mean and the 95% credible intervals of these distributions, respectively. (*c*,*d*) The doubling time, *T*_d_, as a function of the mean delay for oseltamivir and zanamivir, respectively. Black lines show the mean, and dashed red and blue lines show the 50% and 95% credible intervals, respectively.

**Figure 6.**
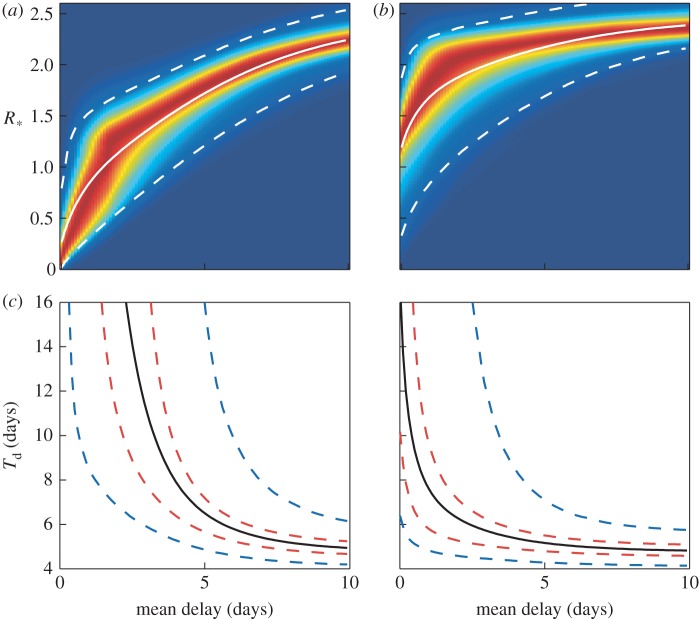
Pandemic model results for the constant delay model. (*a*,*b*) Kernel density plots for the household reproductive ratio, R_*_, as a function of the mean delay to allocation for the two types of antivirals oseltamivir and zanamivir. Solid and dashed white curves in (*a*) and (*b*) mark the mean and the 95% credible intervals of these distributions, respectively. (*c*,*d*) The doubling time, *T*_d_, as a function of the mean delay for oseltamivir and zanamivir, respectively. Black lines show the mean, and dashed red and blue lines show the 50% and 95% credible intervals, respectively.

**Figure 7.**
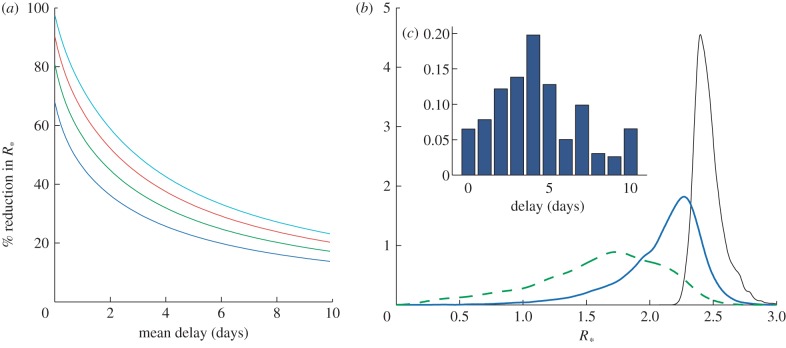
Additional public-health considerations. (*a*) Trade-offs giving a percentage reduction in the household reproductive number R_*_. Here we show the percentage reduction in R_*_ for different combinations of antiviral efficacy and mean delay. We assume the exponentially distributed delay model and pandemic influenza mean parameters, *ρ* = *τ* (blue line, *τ* = 0.5; green line, *τ* = 0.65; red line, *τ* = 0.8; light blue line, *τ* = 0.95. (*b*) Posterior estimates for R_*_ with a delay distribution (*c*) taken from [29]. Kernel density plots are shown for R_*_ assuming no interventions (black curve) and a distribution for the mean delay with zanamivir (solid blue curve) and oseltamivir (dashed green curve).

Figure 5*a*,*b* shows how the densities for R_*_ change with mean delay for the drugs oseltamivir and zanamivir assuming exponentially distributed delays. Figure 5*c*,*d* shows the corresponding change in *T*_d_. Figure 6 shows the same plots, but assuming a constant delay. In the short delay limit (less than 2 days), these tend to the same results as for the exponential delay model. Figure 7*a* shows the percentage reduction in R_*_ for different combinations of antiviral efficacy and mean delay. This allows the exploration of the trade-off between reducing the mean delay and increasing antiviral efficacy. For example, a mean delay of 2 days with an efficacy of 0.5 would give the same percentage reduction in R_*_ as a delay of 4.5 days and efficacy of 0.8. In the absence of more detailed data, we have fixed *τ* = *ρ*, but the trade-off for any range of parameters (and models) could be evaluated in this way. Figure 7*b* shows the posterior distribution for R_*_ with and without interventions using such a delay distribution, taken from Ghani *et al*. [29]. The mean of the distribution is reduced from 2.4 to 1.6, for oseltamivir, and to 2.1 for zanamivir, but there is a large variance in the possible outcomes; this helps to explain the large variation in estimates of R_*_ in the literature [29,33]. Finally, with our mean parameter estimates, we calculated that, on average, 34 per cent of transmission occurs within households, as opposed to externally; this is again in line with previous estimates [16].

## 5. Discussion

We have presented a general modelling framework for studying household-based interventions to combat infectious diseases. This framework was used to study the use of antivirals for prophylaxis during the early stages of an influenza pandemic. In particular, we focused on antiviral effectiveness during the 2009 H1N1 pandemic, and the epidemiological consequences of delays to antiviral delivery.

Our results are relevant to understanding and mitigating pandemics in three key ways. First, it was found that the antiviral efficacies required to stop the invasion of pandemic influenza given expected delays due to notification and subsequent delivery are higher than current estimates. However, antivirals with efficacies as currently estimated [5,6], and with delays which are realistic under a well-planned pandemic response plan [34–36], could have a significant impact on reducing the doubling time in the early stages of the outbreak.

Secondly, our work contributes to the debate on the actual efficacy of antivirals. Ghani *et al.* [29] estimate that the use of the antiviral oseltamivir reduced transmission by 16 per cent at the population level, which is smaller than our estimate of about 33 per cent (using R_*_ as a proxy for overall reduction in transmission). One possible explanation for this discrepancy is that the antivirals are less effective than suggested by controlled trials [25,37], for example, as a consequence of patients not following the correct guidance; a 13 per cent reduction is approximately that estimated for the less efficacious zanamivir. Another explanation is that a more nuanced model is required which takes into account that the effectiveness of the antivirals is a function of the time since initial infection [38].

Thirdly, an extremely robust conclusion of our work is that the main damage due to delayed treatment occurs in the first day or two. This has implications for the trade-offs that must be made in antiviral distribution policy: obtaining early treatment of fewer households, perhaps in combination with targeting of risk groups (such as larger households), may be more efficient than late treatment of a larger number of households. Intuitively, a lengthy delay is likely to mean that the complete household outbreak has run its course before antivirals take effect, mitigating any effect they would have. Note that we would expect this conclusion to be strengthened still further if the reduced biological effectiveness of delayed antivirals were also modelled explicitly, as discussed earlier.

We now turn to methodological conclusions from our work. Here, we have used exponential delays, and also constant delays, to notification and antiviral delivery, which should provide two reasonably extreme cases. This distribution can be replaced with any phase-type distribution, at the expense of an increase in the computational running time of our algorithms; our code is very efficient, and hence there is scope for significant generalization here, and in particular Erlang-2 distributions, for example, could be easily accommodated. Also, as stated earlier, other interventions could be considered, and other epidemiological responses to interventions could also be accommodated. For example, antivirals could also induce an increased recovery rate and their effectiveness could be made to depend on the stage of infection. But, in the absence of detailed information, we have attempted to keep assumptions to a minimum.

Another generalization which could be accommodated within our formulation is different rates of mixing between households of different sizes. Data that would allow for parametrization of such a model are now being collected through large-scale contact surveys [39]. Such a feature in our model is likely to have an impact on the effectiveness of interventions, and may perhaps lead to the identification of optimal targeted intervention strategies. It would also assist in the study of social-distancing interventions, which will influence mixing within and between households in different ways depending, largely, on the household size.

As a final methodological point, we believe the approach adopted for this study of drawing a large number of parameter sets from posterior distributions, and evaluating characteristics for each of these parameter sets, is currently best practise. This allows for kernel density estimates of the full uncertainty in the epidemiological characteristics and is made possible by the computational efficiency of our modelling framework. We hope this approach is adopted more widely in infectious disease modelling studies.
